# A10 RESTORATION OF ARYL HYDROCARBON RECEPTOR SIGNALING IN CELIAC DISEASE BY ORAL TRYPTOPHAN SUPPLEMENTATION: AN EXPLORATORY STUDY

**DOI:** 10.1093/jcag/gwae059.010

**Published:** 2025-02-10

**Authors:** U Kirtikar, G H Rueda, J Szeto, H Galipeau, M Constante, X Wang, M pinto-sanchez, P Bercik, D Armstrong, E Verdu

**Affiliations:** Medicine, McMaster University, Hamilton, ON, Canada; Medicine, McMaster University, Hamilton, ON, Canada; Medicine, McMaster University Faculty of Health Sciences, Hamilton, ON, Canada; Medicine, McMaster University, Hamilton, ON, Canada; Medicine, McMaster University, Hamilton, ON, Canada; Medicine, McMaster University, Hamilton, ON, Canada; Medicine, McMaster University, Hamilton, ON, Canada; Medicine, McMaster University, Hamilton, ON, Canada; Medicine, McMaster University, Hamilton, ON, Canada; Medicine, McMaster University, Hamilton, ON, Canada

## Abstract

**Background:**

Celiac disease(CeD) is an autoimmune disease triggered by the ingestion of gluten in genetically predisposed individuals. The only treatment is a strict gluten-free diet(GFD), however up to 40% of remain symptomatic and have residual inflammation, highlighting the need for adjuvant therapies. We showed CeD patients exhibit impaired microbial metabolism of tryptophan, resulting in reduced activation of the aryl hydrocarbon receptor(AhR), which plays immunomodulatory roles. Increased AhR ligands and activation in the small intestine have been shown in healthy individuals after oral tryptophan, but it is unknown if this supplementation may affect treated symptomatic CeD patients.

**Aims:**

To determine whether tryptophan supplementation improves CeD-specific symptoms, mood symptoms and quality of life in GFD non-responders.

**Methods:**

This double-blinded, placebo-controlled trial aimed to recruit 50 CeD adults on >1 year of GFD with persistent symptoms from McMaster University’s Celiac Clinic. Participants were randomized to L-tryptophan(3g/day) or placebo to be taken daily for 3 weeks. Before and post-intervention, participants completed symptom questionnaires, provided biological samples and underwent endoscopy. The primary outcome was a ≥4-point reduction in Celiac Symptom Index(CSI) from baseline, with secondary outcomes of improvement in Gastrointestinal Symptom Rating Scale(GSRS), Hospital Anxiety and Depression Scale(HADS), and Patient Assessment of Upper Gastrointestinal Disorders-Quality of Life(PAGI-QoL). An interim analysis of first 25 participants was performed to investigate early trends in symptom improvement, expressed as Odds Ratio(OR), 95% Confidence Intervals(CI).

**Results:**

Post-intervention, CSI scores decreased in 67% of participants (8/12) in the tryptophan group compared to 30% (3/10) in the placebo group (*OR=4.36; 95% CI:0.77-28.47*). Similarly, GSRS improved in 75% of participants (9/12) in the tryptophan group vs 40% (4/10) in the placebo group (*OR=4.5; 95% CI:0.73-27.74*). HAD scores improved in 41% (5/12) of the tryptophan group vs 30% (3/10) in the placebo group (*OR 1.6; 95% CI:0.28-9.82*). The PAGI-QoL improved in 58.3% (7/12) of the tryptophan group vs 0% in the placebo group, *p=0.0053*.

**Conclusions:**

A greater proportion of participants under the tryptophan intervention showed improvement in their gastrointestinal, mood symptoms and quality of life compared with the placebo group. We are not currently powered to establish statistical significance for these differences, however, if these results were replicated in the planned study sample size, the study could fulfil its primary outcome. These results encourage to complete recruitment, with further evaluation of the gut microbiota composition and AhR activity.

**Funding Agencies:**

